# COVID-19 Vaccine Hesitancy in a Rural Primary Care Setting

**DOI:** 10.7759/cureus.27196

**Published:** 2022-07-24

**Authors:** Richard Terry, Aeman Asrar, Samantha Lavertue

**Affiliations:** 1 Family Medicine, Lake Erie College of Osteopathic Medicine, Elmira, USA; 2 Family Medicine, Arnot Health, Elmira, USA

**Keywords:** covid-19 vaccine, coronavirus disease 2019, rural area, community health & primary health care research, community survey, vaccine hesitancy, covid-19

## Abstract

As the United States clamors with anti-vax protests, researchers seek to understand what social and behavioral values are keeping patients from electing to vaccinate themselves against the coronavirus disease 2019 (COVID-19) virus. Over the past year, the race to vaccinate has become less about developing working vaccines and more about finding ways to encourage vaccine uptake. This paper examines the question of vaccine hesitancy in rural Chemung County, NY. In identifying various psychosocial barriers to patient vaccination, which we hypothesize will be mostly political, we seek to understand the local mindset in the hope that our data guide the way to change it.

## Introduction

The SARS-CoV-2 virus that resulted in the coronavirus disease 2019 (COVID-19) pandemic was first detected in Wuhan, China, in December 2019. This novel coronavirus spread rapidly around the world, and in March 2020, the World Health Organization (WHO) declared COVID-19 a worldwide pandemic [1]. Current statistics show over 520 million cases worldwide, with more than six million deaths as of May 2022 [2].

Since the first case of COVID-19 emerged over a year ago, the extent of its consequences has been devastating in many ways. The global health, social, and economic effects of this pandemic will be felt for years to come. Before the production of efficacious vaccines, efforts to prevent the spread of COVID-19 relied heavily on social distancing, self-isolation, hand hygiene, mask mandates, travel restrictions, and widespread testing.

As vaccines became available between December 2020 and March 2021, the next step in preventing the spread of COVID-19 became mass vaccination to reach herd immunity, which is defined as a high enough vaccination percentage within the community to prevent the massive spread of disease [3,4]. Vaccines available and deemed effective by the Centers for Disease Control and Prevention (CDC) are Johnson & Johnson, Moderna, and Pfizer [5]. Over 11 billion vaccine doses have been given worldwide; however, in Chemung County, NY, where this study was conducted, the vaccination rate is only 58.99% [6].

Vaccine hesitancy is defined as a motivational state of being conflicted about the effects or safety of a certain vaccine or being opposed to vaccination in general [7]. Vaccine hesitancy is not a single entity; it lies between complete acceptance and refusal of all vaccines. Within the context of this pandemic, the term “anti-vaxer” may apply only to the COVID-19 vaccine rather than as a blanket term for those who refuse all vaccines. In researching the reasoning of the unvaccinated, our terminology must expand with our understanding of their personal barriers [1,8].

The dilemma of COVID-19 vaccine hesitancy is multifactorial and comprises many barriers. Of primary concern is the politicization of vaccine uptake and a growing sense of distrust in the government and mainstream media’s representation of “health facts” on the news [9]. Additional factors include whether to trust the vaccine or the provider of the vaccine. Dror et al. found that geopolitical concerns over the vaccine's country of origin were likely to impact the uptake of the vaccine globally [10]. Moreover, the concerns of Americans include factors such as the speed of the vaccine’s development, its components, efficacy, the rigor of testing, and its potential for causing long-term adverse effects [9]. Many patients also struggle to understand the value of the vaccine and they lack convenient access to it [11].

Many employers under the guidance of New York State, especially within health care, have introduced vaccination mandates for employees to reduce the burden of COVID-19 among healthcare workers. These mandates were an ultimatum: be vaccinated or be unemployed. In many places, mandates were met with hostility and a mass exodus of healthcare workers who stated similar reasons for vaccine distrust as listed above. In rural New York, where our study was conducted, the medical center lost 80 employees to vaccine mandates. Some employees resigned voluntarily before the deadline due to disagreement with the mandate, and those who refused to be vaccinated by the deadline were terminated. These individuals were willing to risk their financial security rather than receive the vaccine, believing that the act of a mandate inherently violates the principle of their autonomy [12]. New York State was dumbfounded by the negative response to these mandates, namely, severe staffing shortages, and in February 2022, decided that despite the original intent of its mandate, the state would not be enforcing the mandate regarding booster shots [13]. This decision was not based on health data but rather a response to social behaviors that dictated the necessity of removing a mandate to staff hospitals amidst the crisis of a pandemic, further illustrating the complexity of questions and problems regarding the vaccination of American citizens. More data on the consequences of vaccination mandates on the attitudes of employees will be required to understand the impact of the concept on global health and the economy in other democratic states.

Anti-vaccine attitudes and beliefs that pre-date the COVID-19 pandemic pose another significant barrier to vaccine uptake along with a pervasive lack of trust in pharmaceutical companies and government agencies such as the CDC. People with conservative political beliefs, residence in non-metropolitan areas, and recent refusal of the seasonal influenza vaccine are other major factors and obstacles to COVID-19 vaccine uptake [7].

Public health awareness about the risks of COVID-19 and the benefits of vaccination needs to be communicated in ways that are easily accessible and understandable within the community [14]. This study is valuable as a means to decipher which types and routes of communication may be useful in targeting the vaccination rate in rural America.

## Materials and methods

This study received IRB exemption and was approved by Arnot Health’s System Review Board and Privacy Committee.

Conducted in November 2021, this study is cross-sectional in nature. A self-administered electronic SurveyMonkey questionnaire, included in the Appendix, was sent via e-mail three times over the course of a two-week period. Each group of emails was sent to the same 1243 patient body to increase the response rate. All patients utilized for this survey had verified emails in the clinic’s electronic medical record. No patient identifying information other than age, gender, and race/ethnicity was recorded. The study targeted patients aged 18 years and above who visited the Eastside Primary Care Clinic in Elmira, NY, between 06/01/2021 and 10/01/2021.

The questionnaire was designed and developed specifically for this study, utilizing only questions deemed necessary for establishing the demographics of the patient base and their opinions regarding vaccination. Participants were briefly informed about the objective of the study and the handling of their personal information. Two research experts independently reviewed and validated the questionnaire, and the final version consisted of three domains: socio-demographic variables, beliefs toward COVID-19 vaccination, and potential barriers that may prevent participants from choosing vaccination. Respondents who refused to get the vaccine underwent further analysis within the questionnaire. These questions consisted of both answer choices about their possible reasons for refusal and a free-form response box. Free-form responses were analyzed individually.

Analysis of all responses was conducted with SurveyMonkey (Momentive Inc., San Mateo, CA) and Microsoft Excel (Microsoft Corporation, Redmond, WA).

## Results

A total of 118 responses were collected from 1243 patients surveyed, yielding a 10% response rate. Of patients, 79% indicated previous acceptance of the vaccine, and 21% indicated that they were not willing to take a COVID-19 vaccine. Of the cohort of 25 individuals indicating they were unwilling to accept a vaccine, 80% were women and 20% were men. Of patients, 60% belonged to the age group of 31-50 years, and 88% were White Americans. A total of 72% had been offered COVID-19 vaccination but refused it, and 28% reported not having been offered vaccination. See Table 1 for the breakdown of respondents by demographics.

**Table 1:** Distribution of unvaccinated respondents based on socio-demographic details (n = 25)

Gender	No. of respondents (% of respondents)
Male	5 (20%)
Female	20 (80%)
Age group	
18-30	4 (16%)
31-50	15 (60%)
51-64	4 (16%)
65+	2 (8%)
Ethnicity	
White American	22 (88%)
African American	1 (4%)
Asian	0 (0.0%)
Hispanic	1 (4%)
Other	1 (4%)
Prefer not to answer	0 (0.0%)

**Figure 1:**
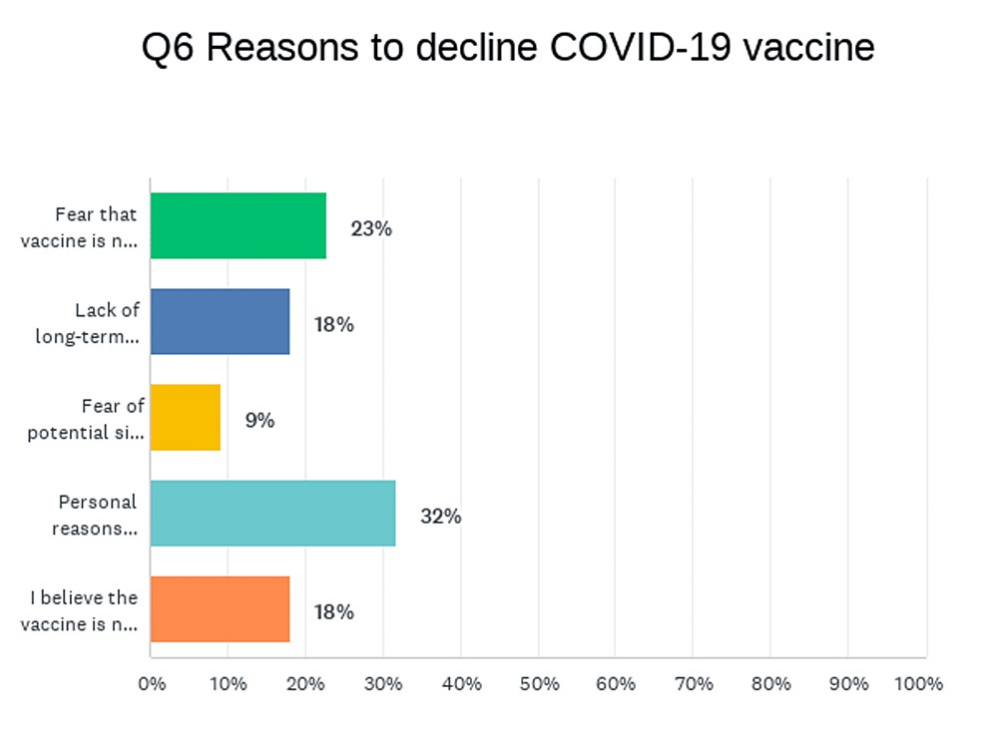
Graphical representation of responses regarding reasons to decline vaccination

When asked to explain their reservations about the vaccine, 23% of respondents said that they feared the vaccine was not safe, 18% were concerned about a lack of long-term safety data, 9% feared potential side effects, 32% disagreed with mandating the vaccine, and 18% believed that the vaccine is not effective. This question, shown in Figure 1, was conducted via choosing the most important reason for vaccine refusal, but patients also had the option to leave comments to elaborate on their choices.

**Figure 2:**
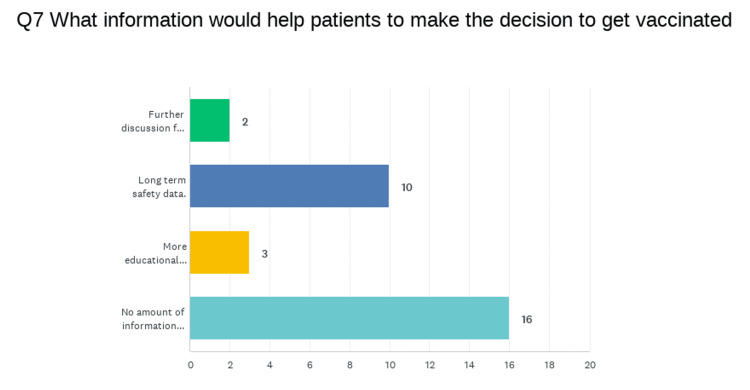
Graphic representation of responses regarding information patients want before choosing vaccination

Figure 2 outlines a question about additional information that could be offered to help patients make the decision to get vaccinated, and within the question, they could select any and all options that they felt applied to their opinion. The box labeled, "no amount of information will change my mind" was selected 16 times. "Long-term safety data" was selected 10 times. "More educational materials" was selected three times, and "further discussion from a health professional" was selected twice.

The free-form responses indicated other lesser reasons for refusing vaccination including pushback against the existence of mandates, concerns that mRNA-based vaccines confront religious beliefs, and personal experiences that they believed to outweigh any clinical data on the subject. One patient responded that she had been hospitalized for COVID-19 and recovered without lasting effects and would “like to see a vaccine that lasts longer than a year.” Another respondent stated, “…whether you receive the vaccine or not, you can still catch COVID!”

Additionally, five patients refused to fill out the survey but emailed us back instead with their negative responses toward the COVID-19 vaccine.

## Discussion

The most cited reason for not getting vaccinated regarded distrust toward the government and governmental policy on COVID-19. In addition, fear about adverse reactions and the rapid vaccination approval process were related to hesitancy regarding vaccination, which is consistent with previous studies. In a report released by the World Health Organization (WHO), congruous, transparent, compassionate, and proactive communication about vaccines was cited to help build trust in COVID-19 vaccines [14]. Avenues such as those discussed in the report may be of benefit to this population of patients with specific fears about the components of the vaccines and their long-term safety.

The key reasons for vaccine refusal in this population were as expected: patients were concerned about the lack of long-term safety data, indignant at mandates, and bothered by the apparent influence of the political sphere on the vaccine’s creation and distribution, a consistent finding within the literature search [8].

The most striking finding of this study was that 66% of unvaccinated respondents said that no amount of information would change their minds about receiving a vaccine, while the remaining participants suggested that more data about long-term safety and provider recommendations could convince them to get vaccinated. A closer examination of these responses indicated pervasive mistrust of the system, which ultimately thwarts the desired outcome of this study: education and increased vaccination rates. It is much easier to collect data and educate a population about the benefits of vaccination than it is to sway the conviction of an individual firm in the belief that taking a vaccine violates his basic rights to autonomy and beneficence. In community settings where these convicted individuals abound, herd immunity becomes less an attainable reality than it is a "pipe dream" of improbability.

This project contains important limitations. With responses from only 118 individuals, of which just 25 were unvaccinated, the small sample size prevents the extrapolation of the data to a larger population. Additionally, several non-vaccinated patients were offended at the mere mention of COVID-19 and chose not to participate in the survey, further limiting responses within the target demographic. While the questionnaire used was validated by an internal team, it was novel and designed solely for the purpose of this study, which limits the ability to compare it to other studies of this type. The questionnaire was also self-administered, and some patients chose to skip questions, which narrows the response rate for portions of the survey and provokes questions about the reliability of self-administered patient surveys.

## Conclusions

The data clearly showed that for one-third of patients, education and personal recommendations from physicians may be enough to convince them to vaccinate, but for the remaining two-thirds of patients vehemently committed against vaccination for personal and political reasons, simple education measures may not be enough to change minds. The questions then become, how do we remove the politicalized stigma of the COVID-19 vaccine, and is non-partisan marketing enough? Where appeals to ethos and pathos have failed, what ultimately motivates the individual to set aside his convictions? Given the rates of unemployment and severe understaffing in health institutions, mandates were not the answer to this question; thus, more studies on a sociological-economic level will need to be completed to answer these questions.

Meanwhile, providers should broach the subject of vaccination with their patients armed with the most current research and a willingness to appeal to what motivates patients on an individual basis. This recommendation requires more of a grassroots effort than can be accomplished with wide-reaching advertisements on television or social media marketing, but it may prove to be a local solution to a local problem.

## Appendices

Informed consent

You are invited to participate in a research study about COVID-19 VACCINE barriers. If you have a moment to spare, please fill out this short survey. It will take less than a minute. The goal of this research study is to identify the barriers and the ways to encourage people to get vaccinated. We’d greatly appreciate your feedback.

This study is being conducted by Dr. Richard Terry, Dr. Aeman Asrar, and Samantha Lavertue (medical student).

Patients who had been to Eastside Primary Care Clinic between 06/01/2021 and 10/01/2021 and 18 years or older qualify to participate in the study.

Participation in this study is voluntary. If you agree to participate in this study, you would be asked to answer some simple questions via an online survey (attached below) about your vaccination status. If you chose not to be vaccinated, we would ask you some questions about why you decided not to receive the vaccine.

Participating in this study will help us learn about attitudes toward COVID vaccination in our community. The questions we would ask you about the decision to not be vaccinated are the sorts of things you might discuss with family or friends.

The information you will share with us if you participate in this study will be kept completely confidential to the full extent of the law. The answers you give to any questions are completely anonymous and will not affect in any way the care you receive.

We will be using SurveyMonkey to administer the surveys. SurveyMonkey will not record your computer’s IP numbers and no patient identifying information other than age and gender will be recorded. Again, your answers will be kept completely anonymous.

If you have any questions about this study, please contact Dr. Aeman Asrar at .

By completing this survey, you are consenting to participate in this study.

We thank you for devoting one minute of your time.

Survey tool

Gender:

* Male

* Female

Age:

* 18-30 years

* 31-50 years

* 51-64 years

* 65+ years

Ethnicity:

* White American

* African American

* Asian

* Hispanic

* Other

* Prefer not to answer

1. Were you offered COVID-19 vaccination?

a. Yes

b. No

If you answered NO to this question, you have completed the survey and do not need to answer any further questions.

2. Did you receive COVID-19 vaccine?

a. Yes

b. No

If you answered YES to this question, you have completed the survey and do not need to answer any further questions.

3. If you were offered vaccination but decided not to get it, please tell us the reason for this decision.

a. Fear that vaccine is not safe because of its rapid development

b. Lack of long-term safety data

c. Fear of potential side effects

d. Personal reasons (disagree with any mandate)

e. I believe the vaccine is not effective

f. Other (please describe) ___________________________

4. What information would you want to receive to help you make the decision to get vaccinated?

a. Long-term safety data

b. More educational material

c. Further discussion from a health professional

d. No amount of information will change my mind

5. Where have you received information about the COVID vaccine? (Check as many as applicable)

a. Print media

b. Broadcast media (television and radio)

c. Internet

d. Word of mouth

6. Do you believe that approved/authorized vaccines are effective at preventing COVID-19?

a. Yes, in most cases

b. No, I don't believe so

c. Yes, but may depend on general health status

7. What strategies do you follow to protect yourself from COVID-19?

a. Wash your hands often with plain soap and water

b. Cover your mouth and nose with a mask when around others

c. Avoid large gatherings and practice social distancing (stay at least six feet apart from others)

d. Nothing

## References

[1] Puri N, Coomes EA, Haghbayan H, Gunaratne K (2020). Social media and vaccine hesitancy: new updates for the era of COVID-19 and globalized infectious diseases. Hum Vaccin Immunother.

[2] (2022). COVID-19 dashboard by the Centers for System Science and Engineering at Johns Hopkins University. https://coronavirus.jhu.edu/map.html.

[3] Hudson A, Montelpare WJ (2021). Predictors of vaccine hesitancy: implications for COVID-19 public health messaging. Int J Environ Res Public Health.

[4] Smith K, Lambe S, Freeman D, Cipriani A (2021). COVID-19 vaccines, hesitancy and mental health. Evid Based Ment Health.

[5] (2022). COVID-19 vaccines are effective. https://www.cdc.gov/coronavirus/2019-ncov/vaccines/effectiveness/index.html.

[6] (2022). Chemung County COVID-19 dashboard. https://forms.chemungcountyny.gov/covidmapupdates/map.php.

[7] Fisher KA, Bloomstone SJ, Walder J, Crawford S, Fouayzi H, Mazor KM (2020). Attitudes toward a potential SARS-CoV-2 vaccine: a survey of U.S. adults. Ann Intern Med.

[8] Jain J, Saurabh S, Goel AD, Gupta MK, Bhardwaj P, Raghav PR (2021). COVID-19 vaccine hesitancy among undergraduate medical students: results from a nationwide survey in India. [PREPRINT]. MedRxiv.

[9] Neely SR, Eldredge C, Ersing R, Remington C (2022). Vaccine hesitancy and exposure to misinformation: a survey analysis. J Gen Intern Med.

[10] Dror AA, Daoud A, Morozov NG (2021). Vaccine hesitancy due to vaccine country of origin, vaccine technology, and certification. Eur J Epidemiol.

[11] Silva J, Bratberg J, Lemay V (2021). COVID-19 and influenza vaccine hesitancy among college students. J Am Pharm Assoc.

[12] (2022). Arnot: 80 employees resign or let-go due to vaccine mandate. https://www.weny.com/story/44917056/arnot-80-employees-resigned-or-let-go-due-to-vaccine-mandate.

[13] (2022). NY won’t enforce booster mandate for health care workers. https://www.adirondackdailyenterprise.com/news/local-news/2022/02/ny-wont-enforce-booster-mandate-for-health-care-workers.

[14] Gatwood J, McKnight M, Fiscus M, Hohmeier KC, Chisholm-Burns M (2021). Factors influencing likelihood of COVID-19 vaccination: a survey of Tennessee adults. Am J Health Syst Pharm.

